# Intra-specific genetic diversity, phylogenetic analysis and ecological preferences of *Pentaclethra macrophylla* Benth*.,* across Nigeria based on *rbcL* dataset

**DOI:** 10.1016/j.dib.2024.110213

**Published:** 2024-02-20

**Authors:** Conrad Asotie Omonhinmin, Kristen Oluwafunmilola Alonge

**Affiliations:** Department of Biological Sciences, Biotechnology Cluster, College of Science and Technology, Covenant University, Canaan land Ota, Ogun State, Nigeria

**Keywords:** *Pentaclethra macrophylla*, African oil bean, Genetic diversity, Evolution, Conservation, Phylogeny, *rbcL* gene

## Abstract

*Pentaclethra macrophylla* Benth., commonly referred to as “African oil bean”; is a leguminous tree species that belongs to the subfamily Caesalpinioideae of the family Fabaceae and it is native to the dry tropical rainforest forest of West to Central Africa. It is widely used as a resource for food, medicine, firewood, construction, arts, and craft and particularly of socio-economic and cultural value to indigenous people of southern, Nigeria. Despite its significant potential, it is considered underutilized in the aspect of research attention and global trade. The dataset highlights the distribution pattern, genetic diversity, phylogenetic relationship, and ecological preferences of *P. macrophylla* accessions collected across the various agro-ecological zones in Nigeria where it is distributed, using the Ribulose 1,5 Bisphosphate Carboxylase/Oxygenase (*rbcL*) gene.

Specifications Table

**Table:** 

Subject	Biological Sciences
Specific subject area	Agricultural, Genetic diversity, Molecular Phylogenetics, Evolution, Environment conditions
Data format	Raw, Analysed
Type of data	Tables, Figures, Repository data
Data collection	Twenty-six (26) leaf samples of *P. macrophylla* were collected across Nigeria, Silica gel dried, and preserved under -80°C (Table 1). All accessions were evaluated using *rbcL* primers. Sequences obtained were edited with Geneious prime, and genetic diversity was determined using state-wise genetic distance on DNA SP. The phylogenetic tree was constructed with MEGA X, codon bias usage, amino acid profiling, and %GC content was determined using DNA SP.
Data source location	The data locations are summarised in Table 1, Figure 1 and GenBank Repository.
Data accessibility	The sequence data of the accessions have been deposited in NCBI GenBank database sequence and has the following accession numbers; OR340863, OR340864, OR340865, OR340866, OR340867, OR340868, OR340869, OR340870, OR340871, OR340872, OR340873, OR340874, OR340875, OR340876, OR340877, OR340878, OR340879, OR340880, OR744875, OR744876, OR744877, OR744878, OR744879, OR744880, OR744881, OR744882. Popset 1 https://www.ncbi.nlm.nih.gov/popset/2557508192[1]. Popset 2 https://www.ncbi.nlm.nih.gov/popset/2616679232[2].

## 1 Value of the Data

• The data provides information on the distribution and genetic diversity of *P. macrophylla* across Nigeria using information from partial *rbcL* gene sequences, nucleotide polymorphism and amino acid composition.
• This data provides information on the amino acid composition and codon usage bias and the %GC composition of the *P. macrophylla rbcL* sequences.
• The data identifies areas of high genetic similarity of *P. macrophylla,* which can be adopted for the improvement of the species, germplasm bank for species conservation and employed for further studies on species.
• The *rbcL* gene sequences can be employed by plant molecular systematists to trace the molecular phylogeny, evolution and possible sub-speciation of *P. macrophylla*.

## 2 Background

The objectives of the dataset are to determine distribution pattern, genetic diversity, phylogenetic relationship, and ecological preferences *Pentaclethra macrophylla* (African Oil bean) in Nigeria.

## 3 Data Description

**Fig. 1:**
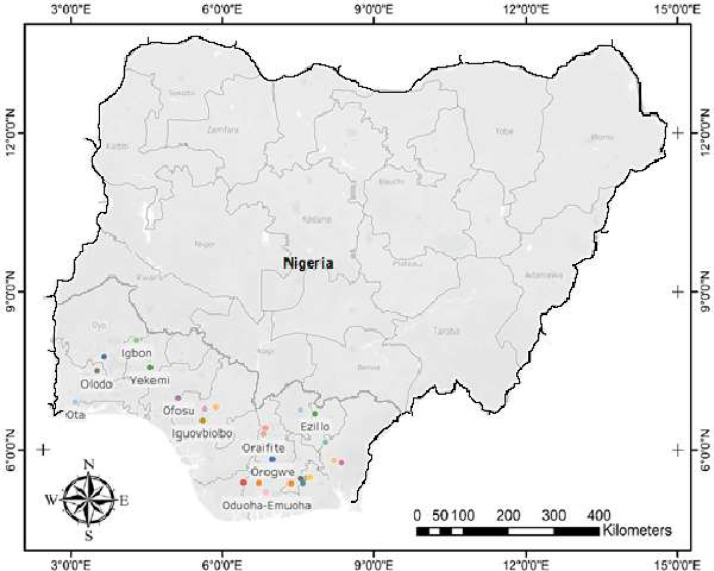
Map showing *P. macrohylla* sample collection areas across the dry rain forest zone of southern Nigeria.

**Fig. 2:**
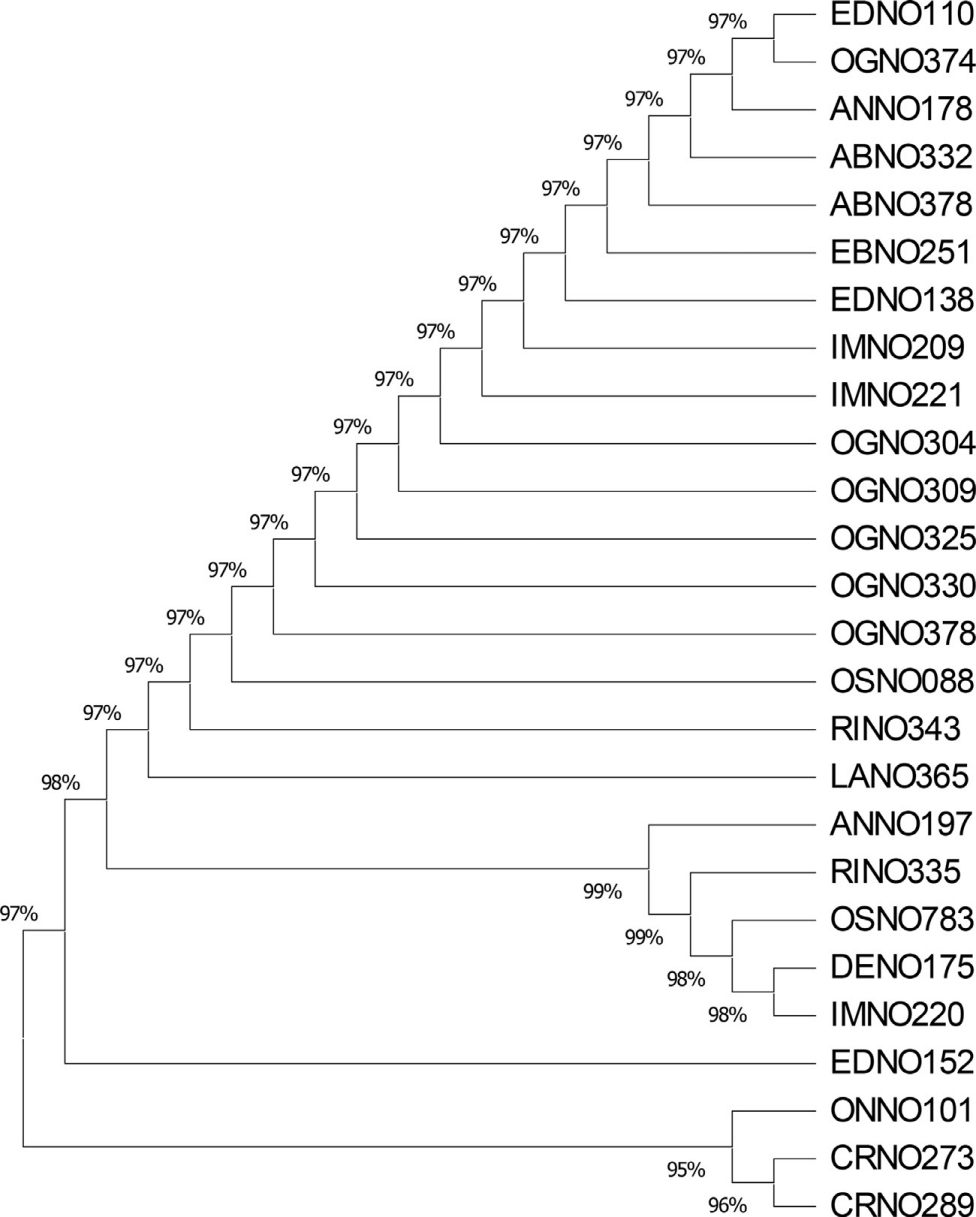
Phylogenetic tree (using Maximum Parsimony method) generated using the *rbcL* sequence data of the 26 *P. macrophylla* accessions.

The data presents the nucleotide sequences of *rbcL* gene for the 26 *P. macrophylla* accessions deposited on NBCI GenBank. Fig. 1; presents the map of the collection sites across the study area [3]. Table 1; list the accessions studied (26), site collection details and NCBI GenBank accession numbers. Table 2; shows the state-wise (within-species) genetic diversity of *P. macrophylla*. Table 3; highlights the nucleotide frequency (TCAG) of the *P. macrophylla* accessions. Table 4; records the amino acid composition of nucleotides *of P. macrophylla* accessions. Table 5; presents the codon bias usage of the *P. macrophylla* accessions as well as their relative synonymous codon usage. Fig 2; presents the phylogenetic tree of *P. macrophylla* accessions.

**Table 1:** Collection Information and geographical co-ordinates of the 26 *P. macrophylla* accessions.

SN	GenBank Accession No	Latitude (^o^N)	Longitude (^o^E)	Altitude (m)	Collection Site	LGA	State
1	OR340863	5.1388	7.526	81	Nlagu	Obingwa	Abia
2	OR340864	4.9005	7.1816	19	Uzuaku	Ukwa West	Abia
3	OR340865	6.0739	6.8192	62	Oba	Idemili South	Anambra
4	OR744875	6.2197	7.0105	140	Enugwu-Agidi	Njikoka	Anambra
5	OR744876	5.9516	8.0295	21	Agbara-Ekureku II	Abi	Cross River
6	OR744877	5.5193	8.1808	126	Betem town	Biase	Cross River
7	OR744878	6.3674	6.4032	207	Idumuja-ugboko	Aniocha North	Delta
8	OR340872	6.2997	6.3773	223	Igbodo	Ika North-East	Delta
9	OR340866	6.4362	7.8101	63	Ezillo	Ishielu	Ebonyi
10	OR340867	6.3834	8.0121	81	Ishieke	Ivo	Ebonyi
11	OR340868	6.4371	5.7824	136	Orokosa	Umagbae-South	Edo
12	OR744879	6.5724	5.9091	135	Erua	Uhunmwode	Edo
13	OR340880	6.4758	7.5509	148	Old Emene Junction	Enugu East	Enugu
14	OR340869	6.0807	7.4701	232	Awgu	Awgu	Imo
15	OR744880	5.5443	6.9763	81	Orogwe	Owerri West	Imo
16	OR340870	5.4312	6.5736	134	Amiri	Otu-East	Imo
17	OR340871	5.7396	6.8838	68	Mgbidi	Otu	Imo
18	OR340873	7.2751	3.5821	169	Olodo	Odeda	Ogun
19	OR340875	7.0537	3.1358	127	Mosan	Oke-Mosan	Ogun
20	OR340876	6.4101	2.5802	2	Owode	Ado-Odo	Ogun
21	OR340874	6.7489	4.9083	108	Ore	Odigbo	Ondo
22	OR340877	7.0059	5.7058	180	Ikaro/Elegbeka	Ose	Ondo
23	OR340878	7.4702	4.363	224	Gbongan	Aiyedaade	Osun
24	OR744882	7.3336	4.5248	298	Erin Oke	Oriade	Osun
25	OR744881	4.5305	6.5527	13	Alakahia	Obio-Akpor	Rivers
26	OR340879	4.8837	6.8616	12	Oduoha-Emuoha	Emohua	Rivers

**Table 2:** Bteween-state genetic distances of *P. macrophylla* accessions.

	Abia	Anambra	Ebonyi	Edo	Imo	Lagos	Ogun	Ondo	Osun	Rivers
Abia	0.000									
Anambra	0.0000									
Ebonyi	0.0000	0.0000								
Edo	0.0000	0.0000	0.0000							
Imo	0.0000	0.0000	0.0000	0.0000						
Lagos	0.0037	0.0037	0.0037	0.0037	0.0037					
Ogun	0.0012	0.0012	0.0012	0.0012	0.0012	0.0049				
Ondo	0.0165	0.0165	0.0165	0.0165	0.0165	0.0165	0.0177			
Osun	0.0000	0.0000	0.0000	0.0000	0.0000	0.0037	0.0012	0.0165		
Rivers	0.0000	0.0000	0.0000	0.0000	0.0000	0.0037	0.0012	0.0165	0.0000	0.0000

**Table 3:** Nucleotide Frequencies of *P. macrophylla* accessions.

Accession	T (%)	C (%)	A (%)	G (%)	%GC
OR340863	29.4	22.0	26.1	22.6	44.2
OR340864	29.4	22.0	26.1	22.6	44.5
OR340865	29.4	22.0	26.1	22.6	44.8
OR340866	29.4	22.0	26.1	22.6	44.5
OR340867	29.4	22.0	26.1	22.6	45.1
OR340868	29.4	22.0	26.1	22.6	44.7
OR340869	29.4	22.0	26.1	22.6	44.9
OR340870	29.4	22.0	26.1	22.6	44.4
OR340871	29.2	22.0	26.2	22.6	44.4
OR340872	29.4	22.0	26.1	22.6	44.8
OR340873	29.4	22.0	26.1	22.6	45.0
OR340874	29.4	22.0	26.1	22.6	44.3
OR340875	29.4	22.0	26.1	22.6	44.8
OR340876	29.4	22.0	26.1	22.6	44.5
OR340877	29.4	21.7	26.6	22.4	47.7
OR340878	29.4	22.0	26.1	22.6	44.6
OR340879	29.4	22.0	26.1	22.6	44.4
OR340880	29.0	21.4	26.5	23.2	45.1
OR744875	29.3	21.5	26.6	22.6	44.0
OR744876	29.5	20.8	27.1	22.6	43.7
OR744877	29.5	20.8	27.1	22.6	43.4
OR744878	29.3	21.8	26.5	22.4	44.2
OR744879	29.9	21.3	26.1	22.4	43.7
OR744880	29.3	21.8	26.5	22.4	44.2
OR744881	29.3	21.5	26.9	22.3	44.0
OR744882	29.3	21.5	26.9	22.3	43.8
AVERAGE	29.4	21.8	26.3	22.5	44.5

**Table 4:** Amino acid composition of *P. macrophylla* nucleotides.

Accession	Ala	Cys	Asp	Glu	Phe	Gly	His	Ile	Lys	Leu	Met	Asn	Pro	Gln	Arg	Ser	Thr	Val	Trp	Tyr	Total
OR340863	8.3	1.7	5.5	6.6	3.9	9.4	1.1	3.9	5.5	9.4	0.6	2.8	7.2	2.2	5.5	3.9	8.3	6.1	1.1	7.2	181.0
OR340864	8.3	1.7	5.5	6.6	3.9	9.4	1.1	3.9	5.5	9.4	0.6	2.8	7.2	2.2	5.5	3.9	8.3	6.1	1.1	7.2	181.0
OR340865	8.3	1.7	5.5	6.6	3.9	9.4	1.1	3.9	5.5	9.4	0.6	2.8	7.2	2.2	5.5	3.9	8.3	6.1	1.1	7.2	181.0
OR340866	8.3	1.7	5.5	6.6	3.9	9.4	1.1	3.9	5.5	9.4	0.6	2.8	7.2	2.2	5.5	3.9	8.3	6.1	1.1	7.2	181.0
OR340867	8.3	1.7	5.5	6.6	3.9	9.4	1.1	3.9	5.5	9.4	0.6	2.8	7.2	2.2	5.5	3.9	8.3	6.1	1.1	7.2	181.0
OR340868	8.3	1.7	5.5	6.6	3.9	9.4	1.1	3.9	5.5	9.4	0.6	2.8	7.2	2.2	5.5	3.9	8.3	6.1	1.1	7.2	181.0
OR340869	8.3	1.7	5.5	6.6	3.9	9.4	1.1	3.9	5.5	9.4	0.6	2.8	7.2	2.2	5.5	3.9	8.3	6.1	1.1	7.2	181.0
OR340870	8.3	1.7	5.5	6.6	3.9	9.4	1.1	3.9	5.5	9.4	0.6	2.8	7.2	2.2	5.5	3.9	8.3	6.1	1.1	7.2	181.0
OR340871	8.3	1.7	5.5	6.6	3.9	9.4	1.1	3.9	5.5	9.4	0.6	2.8	7.2	2.2	5.5	3.9	8.3	6.1	1.1	7.2	181.0
OR340872	8.3	1.7	5.5	6.6	3.9	9.4	1.1	3.9	5.5	9.4	0.6	2.8	7.2	2.2	5.5	3.9	8.3	6.1	1.1	7.2	181.0
OR340873	8.3	1.7	5.5	6.6	3.9	9.4	1.1	3.9	5.5	9.4	0.6	2.8	7.2	2.2	5.5	3.9	8.3	6.1	1.1	7.2	181.0
OR340874	8.3	1.7	5.5	6.6	3.9	9.4	1.1	3.9	5.5	9.4	0.6	2.8	7.2	2.2	5.5	3.9	8.3	6.1	1.1	7.2	181.0
OR340875	8.3	1.7	5.5	6.6	3.9	9.4	1.1	3.9	5.5	9.4	0.6	2.8	7.2	2.2	5.5	3.9	8.3	6.1	1.1	7.2	181.0
OR340876	8.3	1.7	5.5	6.6	3.9	9.4	1.1	3.9	5.5	9.4	0.6	2.8	7.2	2.2	5.5	3.9	8.3	6.1	1.1	7.2	181.0
OR340877	7.7	1.7	5.5	6.6	3.9	9.4	1.1	3.9	5.5	9.4	0.6	2.8	6.6	2.2	5.5	5.5	7.7	6.1	1.1	7.2	181.0
OR340878	8.3	1.7	5.5	6.6	3.9	9.4	1.1	3.9	5.5	9.4	0.6	2.8	7.2	2.2	5.5	3.9	8.3	6.1	1.1	7.2	181.0
OR340879	8.3	1.7	5.5	6.6	3.9	9.4	1.1	3.9	5.5	9.4	0.6	2.8	7.2	2.2	5.5	3.9	8.3	6.1	1.1	7.2	181.0
OR340880	7.7	1.6	5.5	6.6	3.8	11.0	1.1	3.8	6.0	9.3	0.5	2.7	7.1	2.2	4.9	3.8	8.2	5.5	1.1	7.1	182.0
OR748875	8.3	1.7	5.5	6.6	3.9	9.4	1.1	3.9	5.5	9.4	0.6	2.8	7.2	2.2	5.5	3.9	8.3	6.1	1.1	7.2	181.0
OR748876	8.3	1.7	5.5	6.6	3.9	9.4	1.1	3.9	5.5	9.4	0.6	2.8	7.2	2.2	5.5	3.9	8.3	6.1	1.1	7.2	181.0
OR748878	8.3	1.7	5.5	6.6	3.9	9.4	1.1	3.9	5.5	9.4	0.6	2.8	7.2	2.2	5.5	3.9	8.3	6.1	1.1	7.2	181.0
OR748879	7.7	1.7	5.5	6.6	3.9	9.4	1.1	3.9	5.5	9.4	0.6	2.8	6.6	2.2	5.5	5.5	7.7	6.1	1.1	7.2	181.0
OR748880	8.3	1.7	5.5	6.6	3.9	9.4	1.1	3.9	5.5	9.4	0.6	2.8	7.2	2.2	5.5	3.9	8.3	6.1	1.1	7.2	181.0
OR748881	7.7	1.6	5.5	6.6	3.8	11.0	1.1	3.8	6.0	9.3	0.5	2.7	7.1	2.2	4.9	3.8	8.2	5.5	1.1	7.1	182.0
OR748882	8.3	1.7	5.5	6.6	3.9	9.4	1.1	3.9	5.5	9.4	0.6	2.8	7.2	2.2	5.5	3.9	8.3	6.1	1.1	7.2	181.0
**Average**	**8.2**	**1.7**	**5.5**	**6.6**	**3.9**	**9.5**	**1.1**	**3.9**	**5.6**	**9.4**	**0.6**	**2.8**	**7.1**	**2.2**	**5.5**	**4.0**	**8.3**	**6.0**	**1.1**	**7.2**	**181.1**

**Table 5:** Codon Bias Usage of the 26 *P. macrophylla* accessions.

Codon	Count	RSCU	Codon	Count	RSCU	Codon	Count	RSCU	Codon	Count	RSCU
UUU(F)	4	0.99	UCU(S)	4	1.05	UAU(Y)	3	0.51	UGU(C)	5	1.03
UUC(F)	4	1.01	UCC(S)	6	1.59	UAC(Y)	8.8	1.49	UGC(C)	4.8	0.97
UUA(L)	9	4.15	UCA(S)	2.9	0.77	UAA(*)	7.8	1.23	UGA(*)	10	1.59
UUG(L)	0	0.00	UCG(S)	1	0.26	UAG(*)	1	0.18	UGG(W)	6.3	1.00
CUU(L)	2	0.92	CCU(P)	3	1.10	CAU(H)	3.9	2.00	CGU(R)	1	0.28
CUC(L)	0	0.00	CCC(P)	1.9	0.69	CAC(H)	0	0.00	CGC(R)	3	0.86
CUA(L)	2	0.92	CCA(P)	3	1.10	CAA(Q)	4	1.61	CGA(R)	2	0.58
CUG(L)	0	0.00	CCG(P)	3	1.10	CAG(Q)	1	0.39	CGG(R)	3.6	1.04
AUU(I)	6	2.00	ACU(T)	1	0.80	AAU(N)	1.0	0.40	AGU(S)	3.9	1.02
AUC(I)	2	0.67	ACC(T)	2	1.60	AAC(N)	4	1.60	AGC(S)	4.9	1.30
AUA(I)	1	0.33	ACA(T)	0	0.00	AAA(K)	1.4	2.00	AGA(R)	7.3	2.09
AUG(M)	4	1.00	ACG(T)	2	1.60	AAG(K)	0.0	0.00	AGG(R)	4	1.15
GUU(V)	3	2.35	GCU(A)	1	0.68	GAU(D)	0.1	0.07	GGU(G)	1.1	0.54
GUC(V)	0	0.09	GCC(A)	2.9	1.97	GAC(D)	3	1.93	GGC(G)	2	0.99
GUA(V)	1	0.78	GCA(A)	0	0.00	GAA(E)	2	1.33	GGA(G)	1.9	0.95
GUG(V)	1	0.78	GCG(A)	2	1.34	GAG(E)	1	0.67	GGG(G)	3	1.53

Average# codons=181

## 4 Experimental Design, Materials and Methods

### 4.1 Plant material

Twenty-six *P. macrophylla* accessions were collected during a nationwide expedition across 12 states in 2016-2017 (Fig. 1) [3], [4], [5], [6]. The fresh leaf samples of the accessions were silica gel dried, packed in labelled air-tight bags and held at −80°C prior to molecular analysis at the Molecular biology laboratory of the National Institute of Medical Research (NIMR), Lagos, Nigeria.

### 4.2 Genomic DNA extraction

Genomic DNA was extracted using the CTAB protocol [7], and quality and quantity were authenticated using the ThermoFischer^Ⓡ^ Nanodrop spectrophotometer ND-8000-GL

### 4.3 Primer design and synthesis

*P. macrophylla* compatible *rbcL* primers; *rbcL*a_F 5’-ATGTCACCACAAACAGAGACTAAAGC-3’ Forward and *rbcL*a_R 5’- GTAAAATCAAGTCCACCRCG-3’ Reverse; were designed and synthesized for the study.

### 4.4 Gene amplification and DNA sequencing

The PCR amplicons were sequenced at Inqaba Biotec West Africa, Ibadan, Nigeria. Using the *rbcL* primers to amplify a portion of the species chloroplast ribulose 1, 5-bisphosphate carboxylase (*rbcL*) gene.

25 µL PCR cocktail mix was generated for each sample with the following 2.5 µL of 10x PCR buffer, 1.0 µL of 50 mM MgCl_2_, 1.5 µL of 5 pMol forward primer, 1.5 µL of 5 pMol reverse primer, 1.0 µL of DMSO (Dimethyl sulfoxide), 2.0 µL of 25 mM dNTPs, 0.06 µL of NEB OneTaq^Ⓡ^, 2.0 µL of 100 ng/µL DNA (extract), and 13.44 µL of H_2_0.

PCR amplification of the DNA samples was performed on GeneAmp™ PCR System 9700 thermocycler; with initial denaturation at 94°C, 180 sec, 10 cycles; denaturation at 94°C, 15 sec; annealing at 65°C, 30 sec; extension at 72°C, 90 sec, 10 cycles; denaturation at 93°C, 15 sec; annealing at 55°C, 30 sec; extension at 72°C for 90 sec, 30 cycles; and final extension at 72°C, 300 sec. Amplicons were held at 10°C until use [8].

### 4.5 Visualisation of amplicons

Amplified products were loaded on 1.5% w/v agarose gel with 1kbplus GeneRuler DNA Ladders (Thermo Fisher Scientific^Ⓡ^). Gel components ran at 100 volts for 1 hr. The resultant bands were visualised under a UV light trans-illuminator.

### 4.6 Editing and evaluation of DNA sequences and submission

Sequences were cleaned to trim off segments of the sequence that contained stop codons, the best display frames were selected for protein translation. Multiple sequence alignment was performed to generate consensus sequences using the Geneious alignment tool (GENEIOUS Prime 2023.1). The NCBI database reference *rbcL* nucleotide sequence (Popset: 2557508192, 2616679232) was trimmed using the sequencing primers before mapping the sequencing reads to the guide. Using the Geneious alignment tool, generated sequences were individually aligned with the reference *rbcL* sequence after assembly to check for sequencing errors that might result in translation errors. Cleaned lines were then submitted to GenBank using Bankit [9].

### 4.7 Data analysis

Generated *P. macrophylla* accession sequences were aligned to generate consensus sequences using Geneious prime [10]. The default settings were employed for the consensus alignment to obtain the % GC and sequence lengths. The *rbcL* sequences for *P. macrophylla* ranged from 545-908 bp for the accessions. Genetic diversity indices were assessed using DnaSP 4.5 [11], involving the state wise genetic distance. The phylogenetic tree was constructed using MEGA X with the aid of Maximum Parsimony method. The codon usage frequency, amino acid and nucleotide compositions of each *P. macrophylla* accessions sequences were determined using DnaSP 4.5.

## Limitations

The current collection is restricted to Nigeria though the species spread across the dry rainforest of West-Central Africa.

## Ethics Statement

The authors have read and followed the ethical requirements for publication in Data in Brief and confirms that the current work does not involve human subjects, animal experiments, or any data collected from social media platforms.

## CRediT Author Statement

**Conrad Asotie Omonhinmin**: Secure Funding, Conceptualization, Methodology, Supervision; draft review and editing. **Kristen, Alonge**: Experimentation, sequences submission on GenBank, article draft preparation.

## Data Availability

Pentaclethra macrophylla ribulose-1,5-bisphosphate carboxylase/oxygenase large subunit (rbcL) gene, partial cds; chloroplast. (Original data) (NCBI GenBank).
